# MRI Evaluation of Ligamentous Injury in Weightbearing-Stable Suprasyndesmotic Ankle Fractures: A Prospective Observational Study

**DOI:** 10.1177/10711007251352549

**Published:** 2025-07-28

**Authors:** Ola Saatvedt, Håvard Furunes, Peter Franz Schubert, Øyvind Fidje, Marius Molund

**Affiliations:** 1 Department of Orthopedic Surgery, Innlandet Hospital Trust Gjøvik Hospital, Gjovik, Norway; 2 University of Oslo Faculty of Medicine, Oslo, Norway; 3 Division of Orthopaedic Surgery, Oslo University Hospital; 4 Department of Radiology, Østfold Hospital Trust, Sarpsborg, Norway; 5 Department of Radiology and Nuclear Medicine, Oslo University Hospital, Norway; 6 Department of Orthopedic Surgery, Østfold Hospital Trust, Sarpsborg, Norway

**Keywords:** Ankle fracture, deltoid ligament, syndesmosis, stability assessment, weightbearing radiographs

## Abstract

**Background:**

Suprasyndesmotic ankle fractures (Weber C) account for approximately 10% of ankle fractures, and surgery is advised because of the assumed unstable nature of these injuries. Treatment of transsyndesmotic ankle fractures (Weber B) has evolved as weightbearing radiographs are employed as a modality to evaluate ankle joint stability. Joint congruency on weightbearing radiographs indicate sufficient ligamentous integrity to allow for nonoperative treatment. However, no studies have evaluated the ligamentous injury patterns in suprasyndesmotic ankle fractures with a congruent ankle joint on weightbearing radiographs. This study investigates the ligamentous injuries in patients with suprasyndesmotic fractures of uncertain stability that reduce on weightbearing radiographs, aiming to provide further insight into the ligamentous injury patterns of these injuries.

**Methods:**

A prospective cohort study was conducted from October 2023 to August 2024, involving patients with suprasyndesmotic ankle fractures. Eligible patients underwent weightbearing radiographs, and if no medial clear space widening was noted, magnetic resonance imaging (MRI) examination was conducted. MRI results were analyzed for the integrity of the deltoid and syndesmotic ligament complexes to describe ligament injury patterns rather than guide treatment decisions. Weightbearing radiographs at 2-week, 6-week, and 6-12-month follow-up were evaluated for joint congruency and fracture healing. No clinical outcomes were assessed.

**Results:**

Twenty patients were included in the final analysis. The majority of participants exhibited a complete rupture of the anterior inferior tibiofibular ligament and interosseous ligament. Low frequency of complete rupture of the posterior inferior syndesmotic ligament (PITFL) and the deep posterior tibiotalar ligament (dPTTL) of the deltoid complex was evident. The majority of patients demonstrated a congruent ankle joint on the 6-12-month follow-up weightbearing radiographs, with 14 of 15 showing joint congruency. One patient treated nonoperatively demonstrated widening of the medial clear space at the 6-12-month follow-up.

**Conclusion:**

In this small observational study, suprasyndesmotic ankle fractures that demonstrated congruency on weightbearing radiographs often exhibited an intact or partially ruptured PITFL and dPTTL, when evaluated with MRI. Clinical relevance of these findings remains uncertain without outcome data.

## Graphical abstract

**Graphical Abstract:**
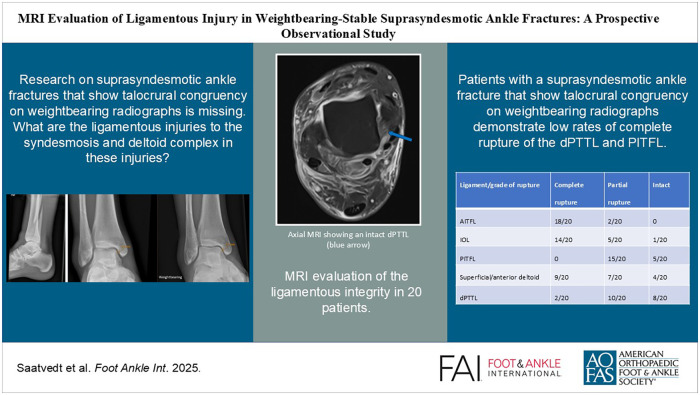
This is a visual representation of the abstract.

**Level of Evidence:** Level III, prospective cohort study.

## Background

Ankle fractures are the most common injuries treated operatively by orthopaedic surgeons, and the incidence is increasing.^15,23^ Suprasyndesmotic ankle fractures comprise around 10% of all ankle fractures, and surgery is widely recognized as the treatment of choice, because of the assumption that these injuries yield instability of the ankle joint.^4,20,26^ According to the work of Lauge-Hansen, suprasyndesmotic fractures occur mainly as a result of pronation type injuries with external rotation.^20^ Such injuries of the ankle commence on the medial side with a fracture of the medial malleolus or a deltoid ligament rupture and subsequently propagate toward the lateral side with injury to the anterior syndesmotic ligament and fibular fracture above the level of the syndesmosis before ending with a posterior syndesmosis ligament rupture or posterior malleolus fracture (Table 1).

**Table 1.** Lauge-Hansen pronation injuries.^20^

Stage	PER
I	Deltoid rupture or medial malleolus fracture
II	AITFL and IOL rupture
III	Suprasyndesmotic fibula fracture
IV	PITFL rupture or Posterior malleolus fracture

Abbreviations: AITFL, anterior inferior tibiofibular ligament; IOL, intraosseous ligament; PER, pronation type injuries with external rotation; PITFL, posterior inferior tibiofibular ligament.

The deltoid ligament complex is the primary stabilizer of the ankle, and advances in the understanding of the anatomy and biomechanics have led to reevaluation of which injuries require surgery and which can safely be treated nonoperatively.^12,13,28^ Several recent clinical studies on Weber B/Lauge-Hansen supination–external rotation fractures emphasize the importance of differentiating what is believed to be partial deltoid ligament tears with an intact deep posterior tibiotalar ligament (dPTTL) from complete tears, as partial tears may be managed nonoperatively if the dPTTL is intact.^8,11,12,14,21^ Furthermore, ongoing research suggests that a subset of suprasyndesmotic fractures may also have an undamaged dPTTL, allowing for nonoperative treatment.^27^ Studies assessing the bony and ligamentous disruptions in this subset of rotational ankle injuries is lacking.

Weightbearing radiographs are now used regularly to guide treatment of trans- and infrasyndesmotic ankle fractures. Patients with a congruent ankle joint on weightbearing radiographs can be treated nonoperatively with satisfactory clinical outcomes.^13,14,17,18^ Weightbearing radiographs are, however, rarely performed on suprasyndesmotic fractures because of the assumed unstable nature of these fractures.

We conducted a prospective cohort study with MRI evaluation of patients with suprasyndesmotic ankle fractures that reduce on weightbearing radiographs with the aim of assessing the ligamentous injuries to the syndesmotic and deltoid ligament complex. There have been several studies using MRI to evaluate the deltoid and syndesmotic ligament complex in acute ankle injuries, but few focusing on suprasyndesmotic fractures and associated ligamentous injury.^1,19^ A deeper understanding of the injury patterns in this understudied subset of suprasyndesmotic ankle fractures may provide further knowledge of the stabilizing ligamentous forces of the ankle joint and aid in diagnostics.

## Methods

### Participants

**Figure 1.**
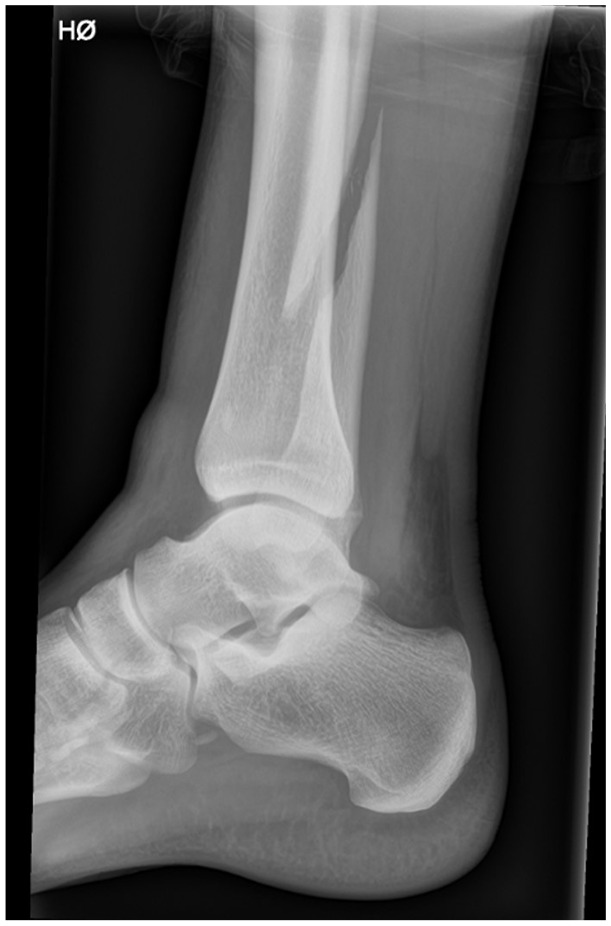
Sagittal plane radiograph showing a suprasyndesmotic ankle fracture.

Eligible patients were prospectively included from Oslo University Hospital, Ullevål and Østfold Hospital Trust, Kalnes from October 2023 to August 2024. Skeletally mature patients (at least 18 years) presenting with a closed fibular fracture classified as suprasyndesmotic with a medial clear space of less than 7 mm in mortise view on initial nonweightbearing radiographs and less than 14 days after injury were considered for study eligibility. A cutoff of 7 mm on nonweightbearing radiographs was implemented to exclude grossly unstable fractures and is in line with previous studies on stability assessment of transsyndesmotic ankle fractures. Patients with Maisonneuve fractures (a fracture to the upper one-third of the fibula), and distal suprasyndesmotic fractures (fractures of the lower two-thirds of the fibula) were included (Figure 1).

**Figure 2.**
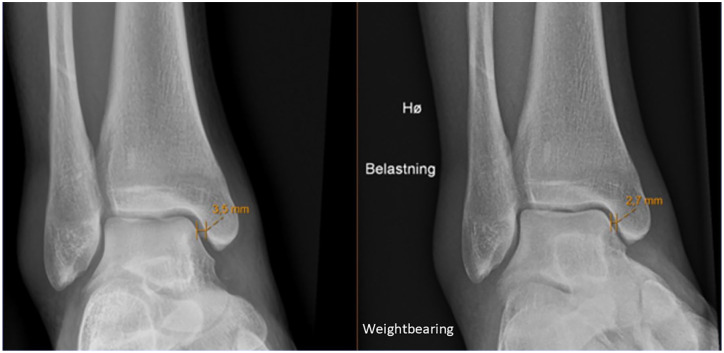
Nonweightbearing (left) and weightbearing (right) mortise view radiographs showing the difference in medial clear space.

Eligible patients underwent a weightbearing (at least 50% of body weight) standing radiograph for the evaluation of the stability of the fracture. Using the uninjured ankle as a control, congruency was considered if the MCS measurement of the injured vs the uninjured ankle was less than 1.0 mm. Patients with a congruent ankle mortise on weightbearing radiographs underwent MRI within 3 weeks of the injury. An orthopaedic, foot and ankle fellowship-trained surgeon measured the medial clear space (MCS) between the medial border of the talus and the lateral border of the medial malleolus on a line parallel to and 5.0 mm below the talar dome in mortise view (Figure 2). Patients with bilateral ankle fractures, a pathologic fracture, a concomitant tibial shaft fracture, a previous fracture, or other notable injuries of either ankle were excluded. Patients who underwent operative stabilization of the injury before MRI were also excluded. All patients were treated according to local clinical guidelines after the MRI examination. Patients were allowed immediate weightbearing as tolerated in an Aircast orthotic device or CAM walker boot. Follow-up appointments with radiographs were conducted at 2 weeks, 6 weeks, and 6-12 months.

### MRI Protocol

All MRI examinations were performed using a 1.5-tesla Siemens Avanto, Amira, or Aera. The patients were examined in the supine position with a neutral ankle position, using a phased-array foot-and-ankle coil. The majority of the patients underwent proton density-weighted fast spin echo imaging. All images were conducted with a 16-cm field of view and 3-mm slice thickness. Sagittal, axial, coronal fat suppression, and coronal oblique T2 images were conducted with a repetition time of 2100 to 3620 milliseconds and echo time of 44 to 97 milliseconds.

### MRI Interpretation

**Figure 3.**
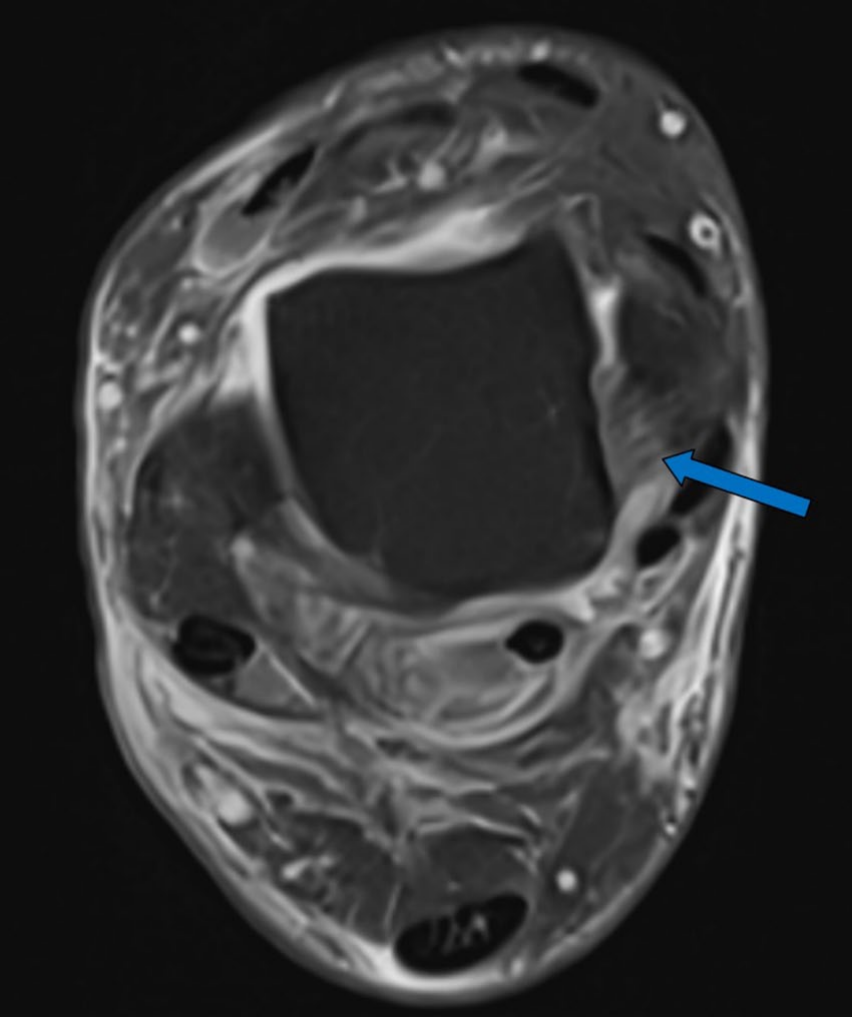
Magnetic resonance image (axial, proton density-weighted fast spin echo) showing marginal signs of edema in an otherwise intact dPTTL (arrow). dPTTL, deep posterior tibiotalar ligament.

**Figure 4.**
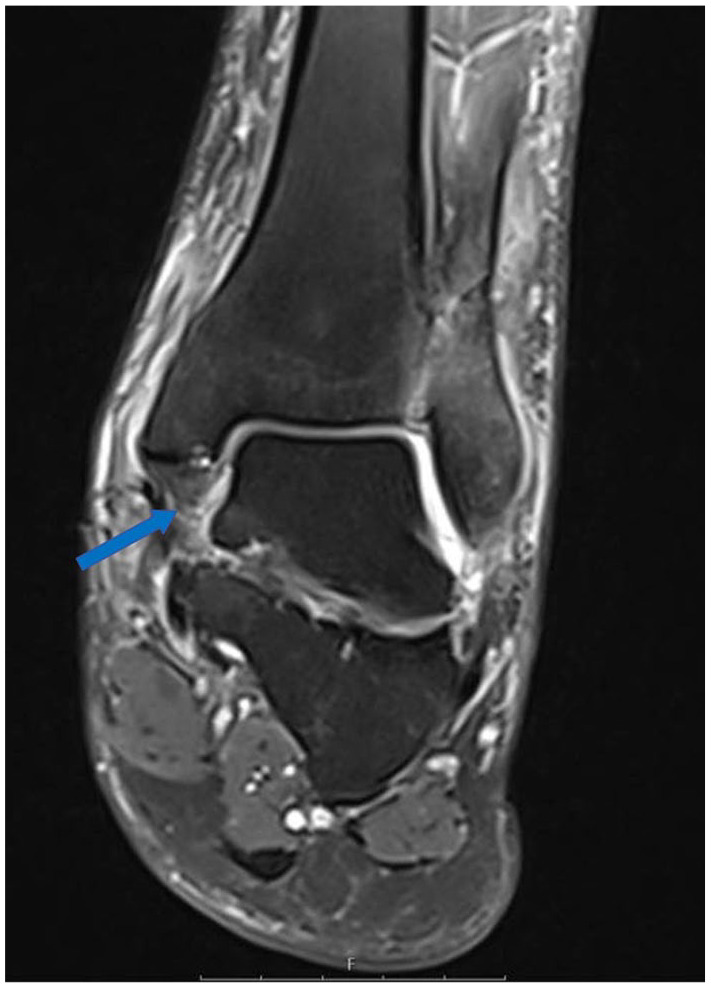
Magnetic resonance image (coronal, proton density-weighted fast spin echo) showing a total rupture of the dPTTL (arrow). dPTTL, deep posterior tibiotalar ligament.

**Figure 5.**
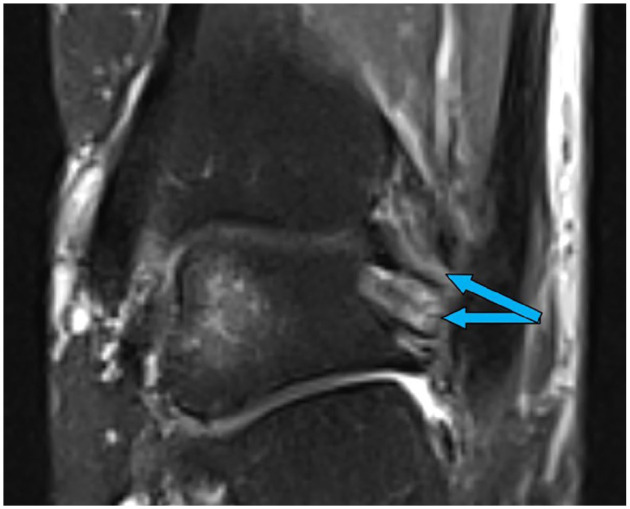
Magnetic resonance image (coronal, proton density-weighted fast spin echo) showing an intact PITFL (arrows). PITFL, posterior inferior tibiofibular ligament.

**Figure 6.**
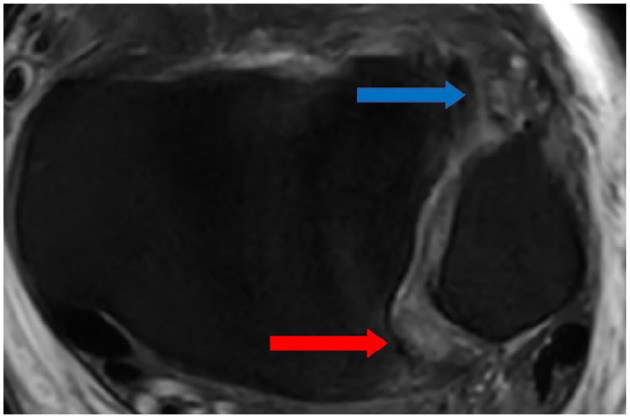
Magnetic resonance image (axial, proton density-weighted fast spin echo) showing a total rupture of AITFL (blue arrow) and partial rupture of the PITFL with edema (red arrow). AITFL, anterior inferior tibiofibular ligament; PITFL, posterior inferior tibiofibular ligament. [See online article for color figure.]

The MRIs were assessed by 2 independent musculoskeletal fellowship-trained radiologists, who were masked to each other’s assessment. In the assessment of the deltoid ligament complex, the dPTTL and the superficial/anterior deltoid were evaluated separately. This separation is supported by previous studies showing the dPTTL as a consistent, well-defined structure (Figures 3 and 4).^6,12^ The superficial/anterior deltoid included the tibiocalcaneal (TCL), tibinavicular (TNL), tibiospring (TSL) and deep anterior tibiotalar ligament (dATTL). The syndesmotic ligaments were divided into the anterior inferior tibiofibular ligament (AITFL), the interosseous ligament (IOL), and posterior inferior tibiofibular ligament (PITFL). The ligamentous integrity was classified as intact, partial tear, or complete tear (Figures 5 and 6). A partial tear was classified as edema and/or discontinuation of some of the ligamentous fibers or a partial avulsion of the ligament insertion. A complete tear was classified as complete disruption of the fibers or total avulsion of the ligament insertion. In cases of disagreement, the final grade was agreed on through discussion. Concomitant injuries such as fractures of the posterior malleolus, Chaput tubercule, or osteochondral injuries were recorded.

### Data Analysis and Statistics

The findings of plain radiographs and MRI were evaluated, and the following data were recorded and analyzed. Variables are summarized as the mean, SD, and range, unless otherwise stated. All analyses were performed with SPSS software (version 21.0; IBM, Armonk, NY).

Mean severity scores were calculated for each ligament assessed on a scale of 1 to 3 (1 = intact ligament, 2 = partial rupture, 3 = complete rupture).

## Results

### Patients

Forty patients were assessed for study eligibility on their primary nonweightbearing radiographs. Of these, 17 patients were excluded because of an MCS greater than 7 mm. One patient showed MCS widening on weightbearing radiographs and was excluded. Twenty-three patients with a suprasyndesmotic ankle fracture that reduced on weightbearing radiographs were originally included. One patient was later recognized as having a transsyndesmotic fracture and was therefore excluded, whereas 2 patients underwent surgical treatment before MRI was performed. The final number of patients used for the statistical analysis were 20 patients. For baseline characteristics and demographics, see Table 2.

**Table 2.** Baseline Characteristics of 20 Patients With Suprasyndesmotic Ankle Fractures Reduced on Weightbearing Radiographs.

Baseline Characteristics	Mean or n (Range)
Age at injury, y, mean (SD)	55 (13)
Sex	
Male	12
Female	8
Injured side	
Left	7
Right	13
Fibular fracture	
Proximal (Maisonneuve)	10
Distal (Weber C)	10
Inclusion center	
Oslo University Hospital, Ullevål	10
Østfold Hospital Trust, Kalnes	10
Treatment	
Operative	3
Nonoperative	17
Medial clear space (mm)	
Nonweightbearing	3.7 (2.5-5.2)
Weightbearing	2.9 (2.1-4.0)
Weightbearing 6-12 mo	3.0 (1.9-5.2)

### Ligament Injuries Graded on MRI

A complete overview of patients and grades of rupture of the respective ligaments is presented in Table 3. Most patients had a total rupture of the AITFL and IOL: 18 of 20 patients (90%) and 14 of 20 patients (70%), respectively. No patients were found to have a complete rupture of the PITFL, but there was a high frequency of partial ruptures (75%). The anterior/superficial deltoid was injured in 16 of 20 patients (80%), with 9 patients demonstrating a complete rupture. The dPTTL was completely ruptured only in 2 of 20 patients (10%). The PITFL and dPTTL were also the ligaments most frequently found intact without any signs of rupture: 5 of 20 patients (25%) and 8 of 20 patients (40%) respectively.

**Table 3.** Frequency and Grade of Injury in Individual Ligaments of the Syndesmotic and Deltoid Complex.

Ligament/Grade of Rupture	Complete Rupture	Partial Rupture	Intact
AITFL	18/20	2/20	0
IOL	14/20	5/20	1/20
PITFL	0	15/20	5/20
Superficial/anterior deltoid	9/20	7/20	4/20
dPTTL	2/20	10/20	8/20

Abbreviations: AITFL, anterior inferior tibiofibular ligament; dPTTL, deep posterior tibiotalar ligament; IOL, interosseous ligament; PITFL, posterior inferior tibiofibular ligament.

The 2 patients with a complete rupture of the dPTTL also had a rupture of the remaining deltoid complex, and 1 patient demonstrated complete rupture of all evaluated ligaments except the PITFL. Four patients were evaluated as having no injury to the deltoid complex, whereas 3 patients demonstrated only injury to the AITFL and IOL. We found no difference in injury patterns between the proximal and distal suprasyndesmotic fractures.

Mean severity scores were calculated for each ligament assessed on a scale of 1 to 3. Regarding the syndesmotic complex the mean (SD) severity score for the AITFL was 2.9 (0.3), IOL 2.7 (0.6), and PITFL 1.8 (0.6). Regarding the deltoid complex, mean severity scores were 2.3 (0.8) for the superficial and anterior deltoid and 1.7 (0.6) for the dPTTL.

### Evaluation of Follow-up Radiographs

**Figure 7.**
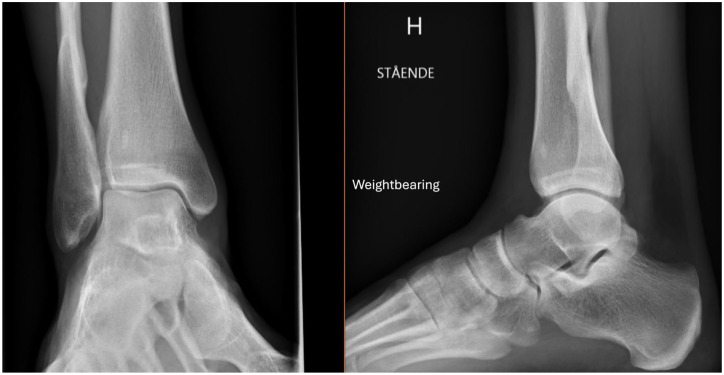
Weightbearing radiographs at the 12-month follow-up showing fracture healing and maintained ankle joint congruency.

One patient was lost to follow-up, and subsequently 19 patients underwent follow-up weightbearing radiographs at 2 weeks, 6 weeks, and 6-12 months after their injury. The ankle joint was evaluated as stable on weightbearing radiographs in 18 patients, demonstrating no medial clear space widening (Figure 7). Of these 18 patients, 3 were treated operatively with syndesmotic screw fixation, whereas 15 patients were treated nonoperatively. One patient showed signs of an unstable ankle mortise with slight MCS widening on weightbearing radiographs at the 6-week and 6-month follow-ups. All fractures showed signs of healing.

## Discussion

In this study, we evaluated the ligamentous injuries of the syndesmotic and deltoid ligament complex in a subset of patients with suprasyndesmotic ankle fractures that reduce on standing weightbearing radiographs. This observational study was not designed to evaluate treatment efficacy or correlate imaging findings with patient outcomes. Studies investigating these injuries are scarce, and very few have challenged their presumed unstable nature. Our results show that most of the pronation type ankle fractures in this study behave as supination external rotation injuries with a fibular fracture, complete rupture of the AITFL and the superficial/anterior deltoid, whereas the PITFL and dPTTL remain intact or only partially damaged. This is in contrast to previous studies that have shown a high frequency of complete disruption of the PITFL and deltoid complex in these injuries.^10,19^ This raises the hypothesis that our approach to the diagnosis and management may potentially be similar to supination external rotation (transsyndesmotic) ankle fractures, though outcome studies are needed to confirm this.

Lauge-Hansen described the cascade in which pronation external rotation injuries occur, but contrary to what Lauge-Hansen concluded in his biomechanical studies we found that parts of the deltoid ligament, namely, the deep posterior tibiotalar, is rarely completely ruptured in patients with weightbearing stable pronation type injuries.^20^ The dPTTL is the strongest entity of the deltoid complex and the largest contributor to tibiotalar stability.^12,21^ A congruent ankle mortise on weightbearing radiographs is mainly due to an intact dPTTL, which tightens in dorsiflexion, centers the plafond underneath the tibia, and resists medial clear space widening.^8,12,21^ The fact that most injuries in our data set have an intact or partially intact dPTTL and PITFL probably explains why they show congruency on weightbearing radiographs. Weightbearing radiographs are used in most Norwegian centers today as a modality to evaluate stability of transsyndesmotic (supination external rotation) ankle fractures and guide treatment

A substantial share of the dPTTL and PITFL were classified as partially damaged with edema and/or discontinuation of certain ligament fibers. There were also cases of thin fracture lines of the posterolateral corner of the posterior malleolus, but with intact periosteum and not affecting the whole insertion point of the PITFL. The integrity and the contribution to stability of a ligament when partially injured is questionable, but it may be expected that they confer more clinical stability compared with a total rupture. The fact that we did not find one patient with a complete rupture of the PITFL might be explained by the extent of deforming forces not reaching the threshold needed to produce a complete rupture, which is thought of as the final stage of a pronation injury cascade. We hypothesize that the external rotation in these injuries is the mechanism in which the dPTTL and possibly the PITFL is spared. Biomechanical studies have shown that external rotation to a degree brings the insertion points of the dPTTL closer together and as a result may spare the ligament from injury.^12^

The radiologists experienced difficulties interpreting the rupture grade of the IOL, especially in proximal fractures (Maisonneuve) as the IOL becomes thin and difficult to distinguish proximally as it forms the distal continuation of the interosseous membrane. Edema in this long segment of the interosseous membrane was at times graded as a partial injury, whereas the distal part showed evidence of a complete rupture. Anatomical and biomechanical studies have demonstrated that the IOL spans from around 1.0 cm above the joint line, extending to the level of the perforating branch of the fibular artery (approximately 4-5 cm above the joint line).^3,16^ In retrospect, we would have limited the height of the evaluation of the IOL to 5 cm above the joint line, and a higher frequency of total ruptures would probably be concluded. As such, it seems that the IOL follows the injury pattern of the AITFL in these injuries, which is in line with previous studies on suprasyndesmotic ankle fractures.^20^

Previous studies on proximal fibular fractures (Maisonneuve) have hypothesized different injury patterns and mechanisms compared with Lauge-Hansen. Pankovich described the mechanism of Maisonneuve fractures in 5 stages, beginning with AITFL and IOL rupture and the deltoid injury or medial malleolus fracture occurring at the final stage.^24^ Some authors have speculated a combination of supination external rotation with concomitant plantar flexion as the basis of these fractures, but no studies to this date have been able to conclude on a well-defined mechanism or staging of injury patterns.^7^ In our material, where 50% of cases were classified as Maisonneuve fractures, we did not find any difference in injury patterns between proximal and distal suprasyndesmotic fibular fractures (see supplementary material).

Operative treatment of suprasyndesmotic ankle fractures is generally regarded necessary to reestablish stability of the ankle joint in order to avoid posttraumatic arthritis. Individual case studies on nonoperative treatment exist, but to date there has not been studies on multiple patients treated conservatively.^5,9,22^ In the presented study, patients were treated according to local guidelines, which employ a similar approach as for supination–external rotation fractures (Weber B). Nonoperative treatment was considered if the patient demonstrated an MCS less than 7 mm on nonweightbearing radiographs, ankle joint congruency on weightbearing radiographs, and a low suspicion of gross instability on clinical assessment. In our study, 15 patients were treated nonoperatively and 1 showed signs of instability on follow-up weightbearing radiographs 6 months after injury (MCS 5.2 mm). This was the only patient in the study who had a complete rupture of all ligaments except the PITFL (partial rupture), and in retrospect one could identify a slight MCS widening on the initial weightbearing radiographs (uninjured: 2.7 mm MCS; injured: 3.5 mm MCS). This highlights the complexity and subtle signs of instability in this patient population and the scrutiny of which the initial evaluation of plain radiographs needs to be based on. Three patients were treated surgically within 3 weeks of injury as a result of shared decision making, after a consultation with an orthopaedic surgeon.

There are several limitations to the present study. This study did not evaluate pain, functional scores, or return to activity. The lack of clinical outcome data limits the ability to interpret the clinical significance of ligament injury patterns seen on MRI. The number of patients is limited because of the relative infrequency of these injuries and the inclusion criteria. Although the study was prospective in nature, treatment decisions (operative vs nonoperative) were made by the treating surgeons and not standardized, introducing potential selection bias. MRI grading relied on 2 expert radiologists and grading of partial vs complete tears—especially of the IOL—may be subject to interpretation bias. In previous studies, the intra- and interrater reliability of MRI evaluation of syndesmotic and deltoid ligaments has been reported to be moderate to good.^2,25^ Notably, the gold standard for evaluating ligament integrity is either with an open or arthroscopic surgery. Few patients in our data set were surgically treated, and we did not have the ethical approval to surgically evaluate ligament integrity. The notion that a congruent talocrural joint on weightbearing radiographs equals global ankle joint stability is a point of current controversy, and one that needs to be further studied. We would expect a higher degree of syndesmotic disruption in pronation-type injuries with external rotation compared with supination–external rotation injuries based on the rotational mechanism, but our study was not designed to evaluate this. Future studies on this subset of suprasyndesmotic injuries should include weightbearing computed tomography to evaluate syndesmotic reduction. Indeed, a randomized controlled trial is currently comparing operative vs nonoperative treatment of these fractures, which may add further evidence of the stability and treatment options in these fractures.^27^

## Conclusion

Patients with a suprasyndesmotic ankle fracture that show no medial clear space widening on weightbearing radiographs demonstrate low rates of complete rupture of the dPTTL and PITFL, when evaluated with MRI. When treated nonoperatively, these fracture types show a tendency to maintain joint congruency when evaluated with weightbearing radiographs at 6-12-month follow-up. Further studies evaluating both clinical outcomes and imaging in this subset of fractures are necessary before altering treatment paradigms.

## Supplemental Material

sj-docx-2-fai-10.1177_10711007251352549 – Supplemental material for MRI Evaluation of Ligamentous Injury in Weightbearing-Stable Suprasyndesmotic Ankle Fractures: A Prospective Observational Study Supplemental material, sj-docx-2-fai-10.1177_10711007251352549 for MRI Evaluation of Ligamentous Injury in Weightbearing-Stable Suprasyndesmotic Ankle Fractures: A Prospective Observational Study by Ola Saatvedt, Håvard Furunes, Peter Franz Schubert, Øyvind Fidje and Marius Molund in Foot & Ankle International

sj-pdf-1-fai-10.1177_10711007251352549 – Supplemental material for MRI Evaluation of Ligamentous Injury in Weightbearing-Stable Suprasyndesmotic Ankle Fractures: A Prospective Observational Study Supplemental material, sj-pdf-1-fai-10.1177_10711007251352549 for MRI Evaluation of Ligamentous Injury in Weightbearing-Stable Suprasyndesmotic Ankle Fractures: A Prospective Observational Study by Ola Saatvedt, Håvard Furunes, Peter Franz Schubert, Øyvind Fidje and Marius Molund in Foot & Ankle International
