# Supplementary material for: MRI Evaluation of Ligamentous Injury in Weightbearing-Stable Suprasyndesmotic Ankle Fractures: A Prospective Observational Study
Source: Foot Ankle Int. 2025 Jul 28;46(10):1131–8. doi: 10.1177/10711007251352549 (PMC12534871; doi:10.1177/10711007251352549)
Supplement: sj-docx-2-fai-10.1177_10711007251352549 – Supplemental material for MRI Evaluation of Ligamentous Injury in Weightbearing-Stable Suprasyndesmotic Ankle Fractures: A Prospective Observational Study [file sj-docx-2-fai-10.1177_10711007251352549.docx]

| Fracture level | AITFL | IOL | PITFL | Superficial/anterior deltoid | dPTTL |
| --- | --- | --- | --- | --- | --- |
| Proximal (Maisonneuve) | Rupture 9  Partial 1  Intact 0 | Rupture 7  Partial 2  Intact 1 | Rupture 0  Partial 7  Intact 3 | Rupture 4  Partial 3  Intact 3 | Rupture 1  Partial 5  Intact 4 |
| Distal (Weber C) | Rupture 9  Partial 1  Intact 0 | Rupture 7  Partial 3  Intact 0 | Rupture 0  Partial 8  Intact 2 | Rupture 5  Partial 4  Intact 1 | Rupture 1  Partial 5  Intact 4 |
